# Human Urine-Derived Stem Cells Originate from Renal Glomerular Parietal Epithelial Cells and Generate Nephrogenic Organoids for Translational Kidney Applications

**DOI:** 10.21203/rs.3.rs-10730001/v1

**Published:** 2026-10-14

**Authors:** Rongpei Wu, Guihua Liu, Yuxin Fan, Nan Deng, Hu Huang, Daniella Beiner, Alan Farney, Anthony Atala, Yuanyuan Zhang

## Abstract

Background Although specialized stem/progenitor cells can be non-invasively isolated from human urine, their precise anatomical origin within the genitourinary tract has remained controversial. Elucidating the cell lineage and tissue niche of human urine-derived stem cells (USCs) is essential to validate and optimize their translational potential. This study aimed to identify the physiological origin of USCs and evaluate their nephrogenic multi-lineage differentiation capacity. Methods Urine specimens were collected from 16 donors, including healthy males, patients with ureteropelvic junction obstruction, and female recipients of sex-mismatched (male donor) kidney transplants. USCs isolated from female recipients were analyzed using fluorescence in situ hybridization (FISH), polymerase chain reaction (PCR), and cytogenetic karyotyping to trace their tissue origin. Surface immunophenotyping was performed via flow cytometry. USCs were evaluated for native marker expression, subjected to directed podocyte differentiation, and cultured in 3D kidney extracellular matrix (k-ECM) hydrogels to assess organoid formation. Results Y-chromosome detection via FISH and PCR in female recipients of male kidney transplants confirmed that USCs originate directly from the donor kidney/upper urinary tract. Flow cytometry and gene expression profiling demonstrated that USCs natively express mesenchymal stem cell and renal parietal epithelial cell (PEC) markers (CD24, CD133, CD44, CD146, ABCG2, PAX2, PAX8, and Claudin-1) while lacking mature podocyte markers (Podocin and Synaptopodin [SYNP]). Directed podocyte induction downregulated PEC markers and induced binucleated podocyte morphology with high SYNP and Podocin expression. In 3D k-ECM hydrogel cultures, USCs organized into tubular structures expressing podocyte (SYNP, E-Cadherin) and proximal/distal tubular epithelial markers (AQP1, AQP3). Conclusions USCs originate directly from renal glomerular PECs and possess robust multipotent capacity to differentiate into both podocytes and renal tubular epithelial cells. These findings establish USCs as a non-invasive, patient-specific renal progenitor source for disease modeling, nephrotoxicity screening, precision diagnostics, and regenerative nephrology applications.

